# Study on Supergenus *Rubus* L.: Edible, Medicinal, and Phylogenetic Characterization

**DOI:** 10.3390/plants11091211

**Published:** 2022-04-29

**Authors:** Qinglin Meng, Hakim Manghwar, Weiming Hu

**Affiliations:** Lushan Botanical Garden, Chinese Academy of Sciences, Jiujiang 332900, China; mengqinglin510@163.com (Q.M.); hakim@lsbg.cn (H.M.)

**Keywords:** *Rubus* L., raspberry fruit, medicinal components, phylogeny, omics

## Abstract

*Rubus* L. is one of the most diverse genera belonging to Rosaceae; it consists of more than 700 species with a worldwide distribution. It thus provides an ideal natural “supergenus” for studying the importance of its edible, medicinal, and phylogenetic characteristics for application in our daily lives and fundamental scientific studies. The *Rubus* genus includes many economically important species, such as blackberry (*R. fruticosus* L.), red raspberry (*R. ideaus* L.), black raspberry (*R. occidentalis* L.), and raspberry (*R. chingii* Hu), which are widely utilized in the fresh fruit market and the medicinal industry. Although *Rubus* species have existed in human civilization for hundreds of years, their utilization as fruit and in medicine is still largely inadequate, and many questions on their complex phylogenetic relationships need to be answered. In this review, we briefly summarize the history and progress of studies on *Rubus*, including its domestication as a source of fresh fruit, its medicinal uses in pharmacology, and its systematic position in the phylogenetic tree. Recent available evidence indicates that (1) thousands of *Rubus* cultivars were bred via time- and labor-consuming methods from only a few wild species, and new breeding strategies and germplasms were thus limited; (2) many kinds of species in *Rubus* have been used as medicinal herbs, though only a few species (*R. ideaus* L., *R. chingii* Hu, and *R. occidentalis* L.) have been well studied; (3) the phylogeny of *Rubus* is very complex, with the main reason for this possibly being the existence of multiple reproductive strategies (apomixis, hybridization, and polyploidization). Our review addresses the utilization of *Rubus*, summarizing major relevant achievements and proposing core prospects for future application, and thus could serve as a useful roadmap for future elite cultivar breeding and scientific studies.

## 1. Introduction

**Figure 1:**
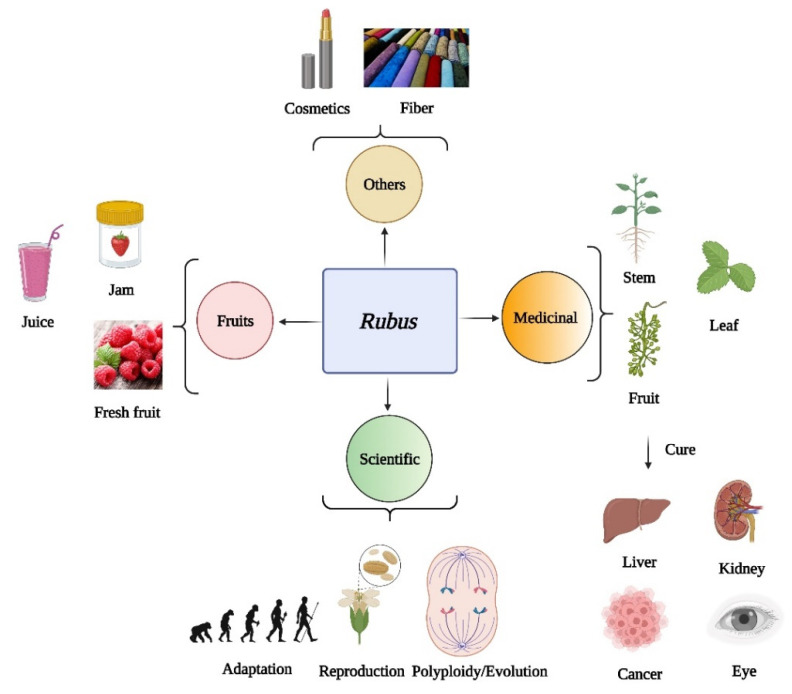
Different aspects of *Rubus* species utilization. The attributes of *Rubus* are primarily utilized for applications involving fruit (fresh fruit, jam, and juice), medicinal compounds (fruit, leaf, and stem), and scientific studies (adaptation, reproduction, polyploidy, and evolution). In addition, among the wild *Rubus* species, some can also be used to produce cosmetics or fiber products. This figure was created using BioRender software.

*Rubus* L. is one of the most diverse and largest genera in the Rosaceae family. The genus consists of more than 700 shrubby or herbaceous species mainly distributed throughout the temperate zone of the northern hemisphere, with a few having expanded to the tropics and the southern hemisphere [1,2,3,4,5,6]. Species of the *Rubus* genus worldwide are classified into 12 subgenera [1,2,3]. However, Lu et al. [6] reclassified them into 8 subgenera, whereby only habitats in China were considered. There are two hypothetical centers of origin for *Rubus*: one is North America [7,8] and the other is southwestern China [9,10,11]. In addition, the pleasant flavor of the fresh *Rubus* fruit, its medicinal functions due to the health benefits of its very high secondary metabolite content, and its high genetic diversity and complex phylogeny rendering it suitable for scientific studies, make *Rubus* an important and ideal genus for breeders as well as scientists [8,12,13] (Figure 1). Furthermore, the rich secondary metabolites and the bark of *Rubus* are also important raw materials for cosmetics and fiber [14].

*Rubus* bears aggregate drupetum fruits that have economically important edible and medicinal characteristics [12,13]. They have a pleasant flavor and have been dubbed “superfoods” due to their very high levels of secondary metabolites, such as hydrolyzable tannins, anthocyanins, polyphenols, flavanols, organic acids, and many other organic compounds [12,15,16,17,18,19,20]. In an early investigation, Moyer et al. [15] extracted multiple anthocyanins and phenols from the ripe fruits of *Rubus*. Based on genomic resequencing, quadrupole time-of-flight liquid chromatography, and mass spectroscopy, 29 hydrolyzable tannins and their candidate chromosomal regions were identified by Wang et al. [20]. Because of these diverse secondary metabolites, the superfood *Rubus* fruits can provide anti-oxidants as well as anti-cancer, anti-microbial, and anti-complement activities, in addition to having other benefits for humans [21,22,23,24,25].

Since the publication of Darwin’s *On the Origin of Species*, understanding the genetic basis of adaptation for the arisal of new species has been a central topic in evolutionary biology [26,27,28,29]. In the species-rich genus of *Rubus*, the diversity of reproductive strategy, such as through the process of hybridization, polyploidization, and apomixis, which enhances the adaptation capacity of *Rubus* [8,30,31]. Additionally, the various reproductive strategies also bring a huge challenge regarding the taxonomy of the *Rubus* genus in terms of morphological and molecular systematics [8,9,31,32,33]. However, in order to make better use of the wild germplasm in *Rubus,* it is necessary to develop a better knowledge of the clear affinity of its phylogeny. Therefore, species of the *Rubus* genus provide a natural experimental system for studying the fundamental mechanisms of adaptation via diverse reproductive strategies and reticulate evolutionary phylogeny.

Over the past hundred years since Focke [1] published *Species Ruborum*, our understanding of *Rubus* L. has improved, including regarding its edibility [4,12,30] and in the medicinal [4,13,15,18,20,34] and phylogenetic fields [7,8,31,35,36] (Figure 1). However, most previous studies and reviews have devoted their attention to metabolic compounds of pharmacologic interest in only a very few specific species [13,19]. This review summarizes the general outcomes for fresh fruit breeding, medicinal components, and studies into phylogenetic relationships of the *Rubus* genus; based on the latest advances in the fields of omics (genomics, transcriptomics, proteomics, and metabolomics), CRISPR/Cas, and other genome editing technologies, experimental efficiency has improved remarkably. Finally, we point out the important value of *Rubus* in fruit germplasms, medicinal research, and understanding complex phylogenetic relationships resulting from diverse adaptative reproduction strategies. In short, *Rubus* L., in the Rosaceae family, is an ideal natural “supergenus” for breeders, pharmacologists, and evolutionary biologists. Our review addresses major questions regarding how to better exploit the wild germplasm in *Rubus* species and can thus serve as a useful roadmap for future breeding and fundamental scientific studies.

## 2. Studies of Edible *Rubus* Species

**Figure 2:**
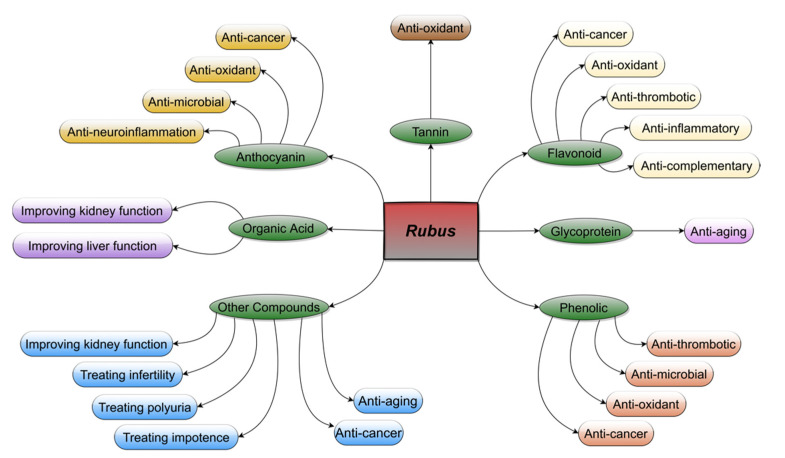
Schematic description of the secondary metabolites of *Rubus* and their medicinal benefits to humans. The central red rectangle represents the wild *Rubus* species, the seven green ellipses represent the major secondary metabolites of *Rubus*, and the outermost ellipses represent the main medicinal functions of secondary metabolites. The various colors show different effects on human health.

*Rubus* L. belongs to the Rosaceae family, from which many palatable fruit cultivars have been bred, such as the woodland strawberry (*Fragaria vesca* L. var. *americana* Porter), and the domesticated apple (*Malus* × *domestica* Borkh.), pear (*Pyrus bretschneideri* Rehd.), and peach (*Prunus persica* (L.) Batsch) [1,9,37]. *Rubus* species are popular for their pleasant fresh fruits, including the blackberry (*R. fruticosus* L.), red raspberry (*R. ideaus* L.), and black raspberry (*R. occidentalis* L.). *Rubus* fruits are aggregate drupetum fruits with varied colors, such as red, yellow, purple, and black [12]. *Rubus* fruits have been called “superfoods” because of the very high levels of beneficial secondary metabolites that they contain, including, e.g., anthocyanins, phenolic acids, flavonoids, tannins, and other essential compounds [19,24,38,39,40] (Figure 2). The chemical composition of *Rubus* is influenced not only by environmental factors but also internal genetic differences. For example, studies of blackberries found that a temperate climate and higher cumulative rainfall increased the production of phenolic compounds [41]. The concentration of chemical components in *Rubus* is also related to the storage conditions, growing season or location, and maturity [42,43,44,45]. The genotype differences between species and cultivars in *Rubus* is the major internal factor that influences the divergence of chemical compositions; for instance, Skrovankova et al. [46] found that different cultivars show significant variations in the production of secondary metabolites, even when they were grown under the same environmental conditions. Furthermore, a series of studies on different *Rubus* genotypes, cultivars, and species produced results consistent with those of Skrovankova [41,44,47,48,49].

Species of the *Rubus* genus have been cultivated and appeared in gardens for more than 15 centuries in Europe, for example in Turkey and Rome [18,38]. To date, thousands of cultivars have been bred, and these can mainly be divided into two types: the primocane-fruiting (also called annual-fruiting) type, including Heritage, Amity, Autumn Bliss, Autumn Britten, Dinkum, and Polana cultivars; and the floricane-fruiting (also called biennial-fruiting) type, including Claudia, Emily, Esta, Lauren, and Qualicum cultivars [8,12,38]. Among these, Logan, Boysen, and Marion are three elite cultivars. The major areas for growing these cultivars are Russia (125,000 t), North America (59,123 t), and Europe (43,000 t) [8,38]. Spurred by the human pursuit of fruit quality and increasing consumption, the fruit production of *Rubus* cultivars has rapidly expanded for the production of fresh fruit for use in jams and fruit juice [12,38,50,51] (Figure 1).

In the early stages of *Rubus* domestication, breeders commonly selected superior individuals from wild habitats [4,5]. During the nineteenth century, approximately 30 breeding projects were conducted in North America and Europe. The elite cultivars of Preussen, Cuthbert, and Newburgh were bred by crossing between different subspecies of red raspberries [30]. However, the process of domestication has vastly reduced the morphological and genetic diversities of crops [52,53,54,55]. Current cultivars are bred from crossing or the improvement of only a few wild species, i.e., red raspberry (*R. ideaus* L.), black raspberry (*R. occidentalis* L.), and blackberry (*R. fruticosus* L.). In order to meet the demands of visual appeal, higher yield, greater quality, excellent health benefits, and diverse adaptation for breeders, more wild germplasms of *Rubus* resources and advanced biotechnology should be utilized for *Rubus* breeding. Based on simple sequence repeat (SSR) and amplified fragment length polymorphism (AFLP) markers, the first linkage map for *Rubus* was constructed based on the crossing of two red raspberry cultivars, Glen Moy and Latham, from Europe and North America, respectively [56]. Bushakra et al. [57] constructed a genetic linkage map using 1218 markers by the crossing of S1 (*R. occidentalis* L.) and Latham (*R. idaeus* L.) and compared it with genomes of other genera in the rosa family, such as *Fragaria* L., *Malus* Mill. and *Prunus* L. That study reported a high consistency of collinearity of genomes between different genera of Rosaceae, and hundreds of new polymorphic genetic markers were found for future quantitative trait loci mapping studies. In recent years, different high-resolution markers and advanced sequencing methods have been applied for trait mapping or new wild germplasm identification in *Rubus* [58,59,60,61,62].

## 3. Medicinal Studies of *Rubus*

*Rubus* L. is one of the most species-rich genera in the Rosaceae family, but only a few species have been used as medicinal herbs [13,18,63]. According to the records of the ancient pharmacopoeias in Europe and China, *Rubus* species have been used as medicinal herbs for several centuries. The stems and leaves of blackberry (*R. fruticosus* L.) were soaked with white wine for use as an astringent poultice for wound healing and for difficulties during childbirth, as suggested by Hippocrates [18]. The dried unripe fruits of “Fu-Pen-Zi” (*R. chingii* Hu) were used to improve and enhance liver and kidney health [20,64]. More recently, as shown in Figure 2, many kinds of secondary metabolites with remarkable beneficial effects on humans have been extracted. Table 1 exhibits detailed information on the species, concentration, and part from which they were extracted. For instance, anthocyanins with an anti-oxidant function are extracted from the fruits of the blackberry [15,19]; flavonoids with anti-oxidant, anti-cancer, and anti-inflammatory effects are extracted from the fruits of the blackberry or Fu-Pen-Zi [16,65,66,67]. Other organic compounds have been identified, mainly in blackberry or Fu-Pen-Zi, such as hydrolyzable tannin, glycoprotein, organic acid, and phenolic compounds [19,21,61,68,69]. In addition, reports have indicated many other kinds of beneficial effect of these secondary metabolites on humans, for example, improving mitosis and eyesight, treating or preventing cancer, back pain, and frequent urination [23,25,70,71]. To date, most of the secondary metabolites mentioned above are considered safe according to data from limited studies [13,72,73,74,75,76]. For example, the extracted components from *R. niveus* Thunb. showed no statistically significant toxicity for mice [72]. Based on a cytotoxic experiment on Caco-2 cells, Ke et al. [77] found that metabolites extracted from the fruit of *R. chingii* Hu were safe and had a favourable effect on anti-cancer cells. Overall, the limited available data on the toxicity and allergenicity of *Rubus* species indicate they are safe for humans. More in-depth investigations regarding medicinal applications in pharmacology are needed.

In the genomic age, genomic sequencing of *Rubus* is already lagging behind compared to other major crops and fruits, such as rice, maize, cotton, apple, and pear [53,78,79,80,81,82,83,84,85]. However, benefiting from the quick development of the cost-effective next-generation sequencing (NGS) [86,87] and transcriptome (RNA-seq) technology [88,89], nuclear or plastid genomes have been sequenced for some important medicinal species in the *Rubus* genus [20,90,91,92,93,94,95]. Utilizing the RNA-seq data of the red raspberry (*R. idaeus* L.) fruit, Hyun et al. [90] determined the regulated candidate genes for biosynthesis of γ-aminobutyric acid and anthocyanins, which have anti-oxidant activity. Based on unripe Fu-Pen-Zi (*R. chingii* Hu) fruits, the chromosome-scale reference and genomic regions related to the biosynthetic pathway for hydrolyzable tannin (HT) have been reported [20]. Therefore, using the results of these studies, breeders could modify candidate genes or genomic regions of *Rubus* cultivars to improve the content of targeted secondary metabolites (HT, anthocyanins, etc.) using site-directed genome editing technologies such as CRISPR/Cas. More recently, in the field of crop breeding, CRISPR/Cas genome-editing technology has been used with encouraging results [96]. However, use of this speedy, proven, and precise genome editing technology has not been reported in programs for breeding *Rubus*. To date, various studies taking advantage of transcriptomic analysis at different developmental stages (green; green and yellow; yellow, orange, and red) of Fu-Pen-Zi fruits have revealed that flavonoids and anthocyanins are synthesized at an early stage and their levels then decrease during subsequent development [61,97,98]. These studies also indicated that anthocyanins might not be responsible for the reddish color of ripe fruits. Thus, better characterization will require extraction of the pharmacological metabolites at the early stage of *Rubus* fruit development. Meanwhile, several studies on plastid genomes have focused on the pharmacological components of *Rubus*, i.e., *R. eucalyptus* Focke [93], *R. rufus* Focke [99], *R. longisepalus* Nakai and *R. hirsutus* Thunb. [100,101], and *R. phoenicolasius* Maxim. [94]. However, the detailed genetic basis and biosynthetic pathways of the pharmacological metabolites in *Rubus* species are still largely unclear, and further future investment and research on different aspects are needed [102,103,104].

**Table 1:** Major secondary metabolites of *Rubus* L.

Secondary Metabolite	Species	Concentration * (mg/100 g)	Part	References
Anthocyanin	*R. chingii*	2.1~326	Leaf	[20,39,40,105,106]
	*R. fruticosus*		Fruit	
	*R. ideaus*			
	*R. hirsutus*			
Flavonoid	*R. chingii*	2.8~6	Leaf	[20,34,40,107]
	*R. occidentalis*		Fruit	
Phenolic compounds	*R. chingii*	13.7~1541	Root	[19,20,39,40,48,105,106,108]
	*R. occidentalis*		Stem	
	*R. setchuenensis*		Leaf	
			Flower	
			Fruit	
Organic acids	*R. chingii*	0.2~52.9	Stem	[20,109]
	*R. coreanus*		Leaf	
			Fruit	
Glycoprotein	*R. chingii*	14.6~81.4	Fruit	[63]

* The concentration data were collected from multiple studies in which the extraction method, part, and species varied, and thus the data presented in the table show minimum and maximum values.

## 4. Phylogenetic Studies of *Rubus*

The *Rubus* genus is one of the most successful models of an adaptive and evolutionary group, with distribution worldwide except for Antarctica [1,9]. As shown in Table 2, *Rubus* species were classified by Focke into 12 or by Lu into 8 subgenera according to worldwide distribution or distribution in China, respectively. The classification and phylogenetic construction of *Rubus* is a challenging task due to phenomena such as hybridization, apomixis, polyploidization, and introgression, which happen frequently in this genus. Ploidy levels among different subgenera and species are highly differentiated [7,32,33,35,58] (Table 2). More importantly, the diverse reproductive strategies may have conferred to *Rubus* species the ability to occupy various habitats worldwide, and thus demonstrate reticulate evolutionary phylogeny. According to previous phylogenetic analysis based on the *ndhF* gene, Howarth et al. [110] suggested that the Hawaiian Islands species (*R. hawaiensis* A. Gray and *R. macraei* A. Gray) originated from different ancestors, in contradiction with the morphological results. The reticulate evolution of *Rubus* has been indicated in recent studies. Wang et al. [31] used multiple chloroplast and nuclear genes to investigate the phylogenetic relationships of 142 *Rubus* taxa, which indicated reticulate evolutionary events between different subgenera and species. A study based on approximately 1000 target genes constructed the phylogenetic tree for 87 wild *Rubus* taxa and three cultivars, concluding that hybridization and incomplete lineage sorting (ILS) were responsible for the low resolution and topological conflicts between different subgenera, which were not caused by insufficient molecular signals [8]. Furthermore, it has been suggested that North America might be the primary center of origin of *Rubus*, which then expanded into Asia and Europe and finally dispersed to Oceania via birds [8].

Additionally, a series of studies on the phylogeny of *Rubus* detected conflicts in the phylogenetic affinities between plastid genes and nuclear genes in most cases [36,114,115]. The molecular and morphological topotaxies also appear inconsistent, which may result from the multiple reproductive strategies [36,111,114,115,116,117]. In general, the difficulties of morphological or molecular taxonomy in subgenera and between species are not caused by lack of characteristics or signals; the real reason may be the diverse reproductive patterns that have made this group an ideal genus for investigation of the genetic basis of different reproductive and adaptive patterns.

## 5. Concluding Remarks and Future Perspectives

The *Rubus* genus consists of more than 700 species, but only a few of them, such as blackberry (*R. fruticosus* L.), red raspberry (*R. ideaus* L.), and black raspberry (*R. occidentalis* L.), have been domesticated or crossed by breeders to generate elite cultivars with excellent characteristics of strong adaptability, good storage properties, and pest or disease resistance. Unfortunately, there have been relatively few molecular breeding studies on *Rubus* and fewer genomic resources exist compared to other types of crops and fruits. *Rubus* breeders can reference those studies in order to improve the breeding methods for elite cultivars of different crops and fruits.

Furtehrmore, the situation for medicinal cultivars of *Rubus* is worse, and most of the investments in pharmacology have been concerned with *R. ideaus* L., *R. fruticosus* L., and *R. chingii* Hu, rarely involving residual species such as *R. eucalyptus* Focke, *R. occidentalis* L., and *R. phoenicolasius* Maxim., which have also been used as medicinal ingredients for hundreds of years. Notably, there remains a large deficiency in the study of the basic mechanisms and genetics of the active ingredients in medicinal *Rubus* species. Fortunately, advances in NGS and RNA-seq technologies offer an opportunity for researchers to spend less money and labor investigating the above-mentioned problems and to quickly identify and choose high-quality germplasms.

Finally, reconstructing the phylogenetic relationships for *Rubus* is a task made challenging by hybridization, polyploidization, apomixis, and introgression. However, researchers can also combine consideration of morphological characteristics with omics technologies (i.e., genomics, transcriptomics, proteomics, and metabolomics) to decipher the phylogenetic and evolutionary puzzles of *Rubus*. Consumers in the present era are increasingly demanding tastier and healthier fresh fruits. The wild species and elite cultivars of *Rubus* provide ideal candidates to address this demand due to their pleasant flavor and high concentrations of secondary metabolites. However, only a few wild species have been domesticated and are used in our daily food markets and medical treatment. Therefore, in order to better exploit the abundance of *Rubus* wild germplasms, information on their phylogenetic relationships and genetic diversity should be clarified. This study reviewed three major topics (edible, medicinal, and phylogenetic properties), but many challenges still exist in the utilization and research of *Rubus*. Working as a team and applying the latest omics strategies may open the door for developing a series of satisfactory elite germplasms for fruit and medicine, and reveal the central evolutionary phenomenon resulting in reticulate evolution.

## Figures and Tables

**Table 2:** List of *Rubus* L. subgenera.

Subgenus	Code	Species in Subgenus	Ploidy Level (*x* = 7)	References
*Anoplobatus*	*An*	9	2*x*	[8,30,111,112]
*Chamaebatus*	*Cb*	6 (5)	2*x*, 6*x*	[8,30,31]
*Chamaemorus*	*Cm*	1 (1)	6*x*, 8*x*	[8,30]
*Comaropsis*	*Co*	2	4*x*	[8,30]
*Cylactis*	*Cy*	18 (8)	2*x*−4*x*	[8,30,31]
*Dalibarda*	*Da*	5	2*x*	[8,30]
*Dalibardastrum*	*Ds*	15 (10)	4*x*, 6*x*	[8,30,31,111,112,113]
*Idaeobatus*	*Id*	125 (83)	2*x*, 3*x*, 4*x*, 13*x*, 18*x*	[8,20,30,31,111,112,113]
*Lampobatus*	*La*	10 (1)	4*x*	[8,30]
*Malachobatus*	*Ma*	104 (85)	4*x*, 6*x*, 8*x*, 14*x*	[30,31,111,112,113]
*Orobatus*	*Or*	16	6*x*	[8,30]
*Rubus*	*Ru*	444 (1)	2*x*−12*x*	[8,30,111,112]

The number within brackets is the corresponding number of *Rubus* species in China.

## Data Availability

Data sharing not applicable.
